# Supplementary material for: HitoMi-Cam: A Shape-Agnostic Person Detection Method Using the Spectral Characteristics of Clothing
Source: J Imaging. 2025 Nov 7;11(11):399. doi: 10.3390/jimaging11110399 (PMC12653039; doi:10.3390/jimaging11110399)
Supplement: Supplementary file 1 [file jimaging-11-00399-s001.zip › jimaging-3951176-supplementary.pdf]

Supplementary Material for
HitoMi-Cam: A Shape-Agnostic Person Detection Method Using the Spectral Characteristics of Clothing

Shuji Ono

Supplementary Figures

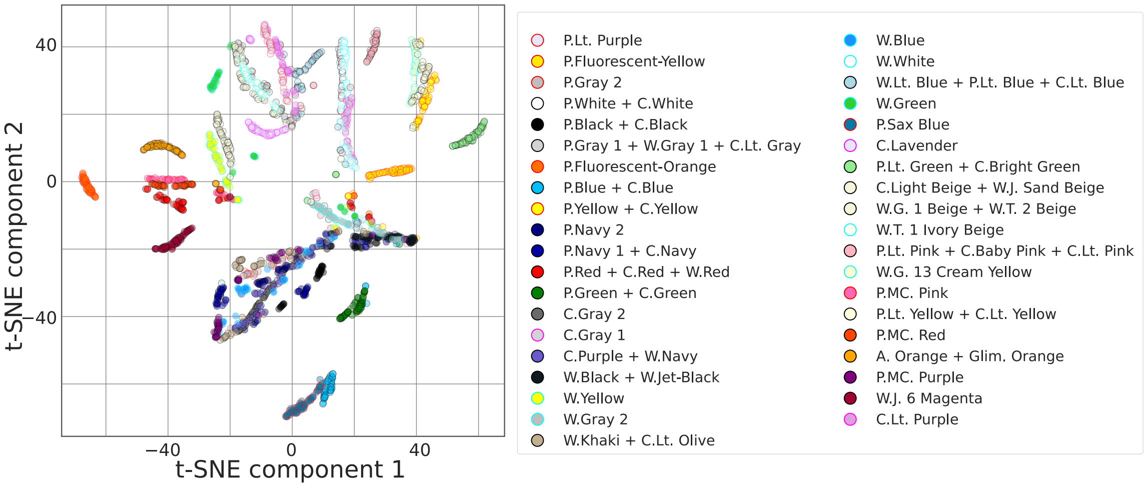

Figure S1: t-Distributed stochastic neighbor embedding (t-SNE) visualization of hyperspectral signals for 39 labeled clothing categories. Each point represents a clothing sample, plotted in a two-dimensional space based on its 167-band hyperspectral signal. The distribution illustrates the spectral diversity of the clothing samples used in the training dataset. This figure is adapted from the author's previous work.

[1] Key to abbreviations: P. = polyester, C. = cotton, W. = wool, T. = Toray (a Japanese synthetic fabric manufacturer), G. = gabardine, MC. = mixed color, J. = Josette (a linen-based fabric), A. = athletic, Glim. = glimmer, and Lt. = light.

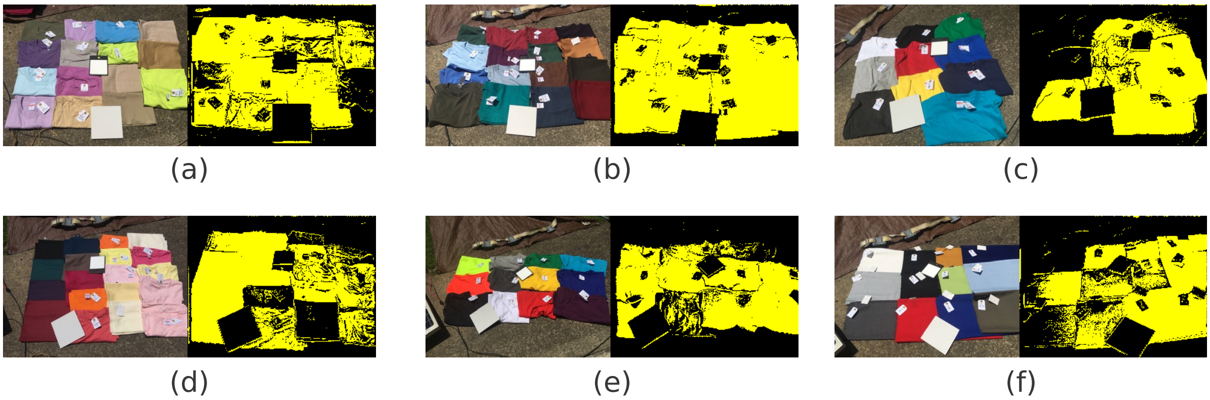

Figure S2: Example of results of basic verification experiment with *HitoMi-Cam* prototype. Representative examples of the 84 types of clothing samples whose spectral characteristics were measured in the previous study were photographed under natural outdoor light to verify the detection performance. (a)–(g) show the detection results for each sample, with the yellow area indicating the pixel map correctly detected as ”clothing” by *HitoMi-Cam*. Of the 84 types, 71 were correctly detected (a recall rate of 84.5%), demonstrating that the detection principle based on spectral information shown in the simulation is physically reproduced by the constructed hardware.

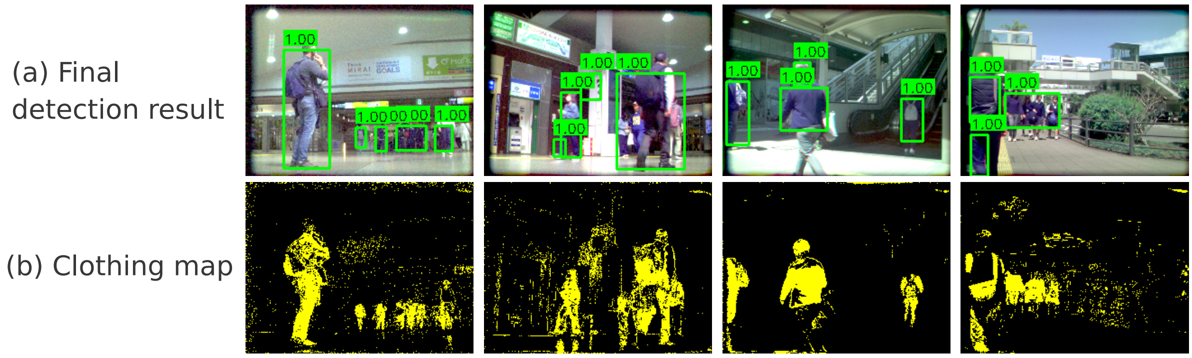

Figure S3: Qualitative results of *HitoMi-Cam* in the General Scene. (a) Examples of final detection results. (b) The corresponding examples of intermediate clothing maps.

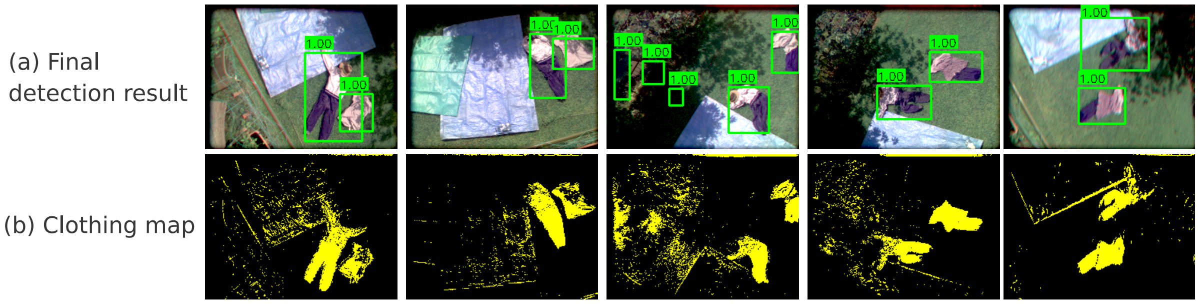

Figure S4: Qualitative results of *HitoMi-Cam* in the Simulated SAR Scene. (a) Examples of final detection results. (b) The corresponding sequence of intermediate clothing maps. The examples include various challenges, such as complex backgrounds (vegetation, blue tarp) and lighting (shade). Notably, the rightmost example demonstrates robustness to significant motion blur, a condition that typically degrades the performance of shape-based detectors.

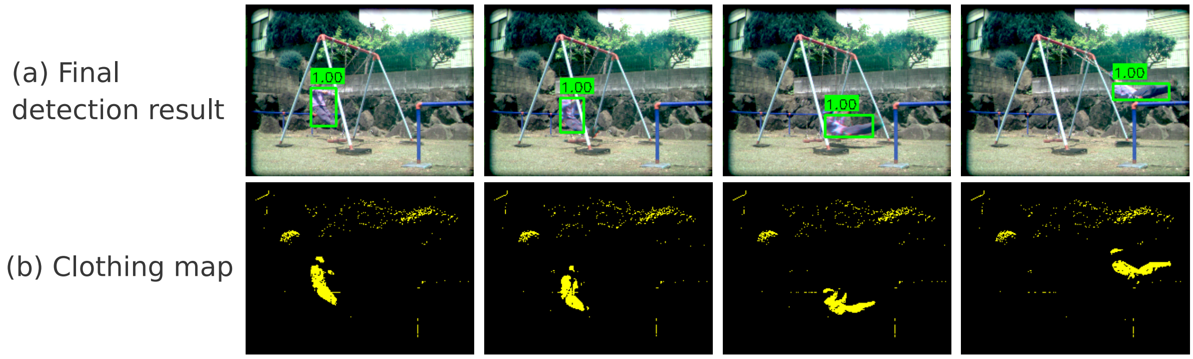

Figure S5: Qualitative results of *HitoMi-Cam* in the Swing Scene. (a) A sequence of final detection results. (b) The corresponding sequence of intermediate clothing maps.

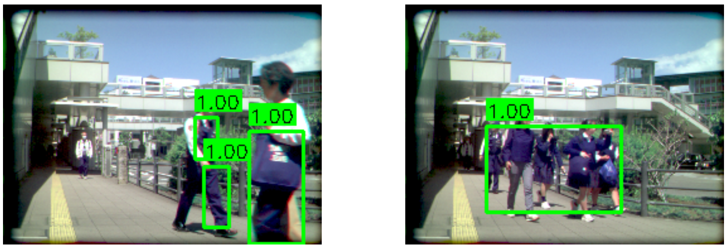

(a) Partial detection (b) Merging detections

Figure S6: Analysis of *HitoMi-Cam*'s failure cases for General Scene. This figure illustrates typical failure modes of *HitoMi-Cam* in crowded public scenes. (a) Partial detection: Due to spectral limitations, only the upper body clothing is detected, resulting in a bounding box that is too small. (b) Merging detection: The algorithm incorrectly merges five closely located individuals into a single detection, failing to distinguish them as separate entities. These examples clarify the reasons for the lower AP score in the General Scene scenario.

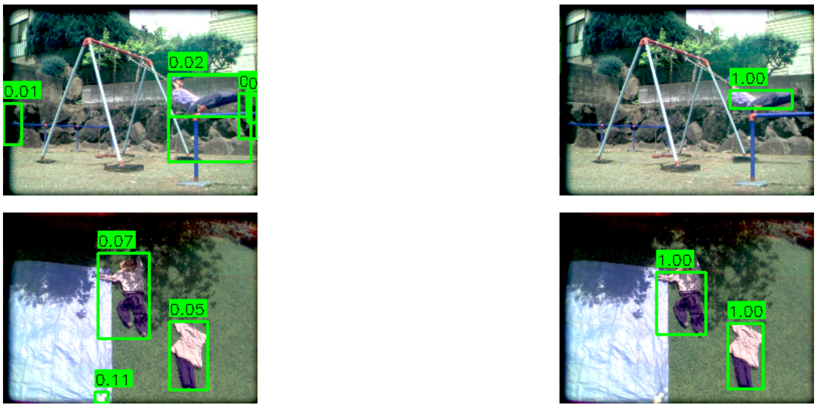

(a) YOLOv5x fails (b) HitoMi-Cam detects

Figure S7: Qualitative comparison with baseline detector (YOLOv5x) in challenging cases. While the baseline method (a) fails, *HitoMi-Cam* (b) successfully detects the target. These examples highlight the robustness of *HitoMi-Cam* against non-human shapes (from Simulated SAR Scene) and significant motion (from Swing Scene).

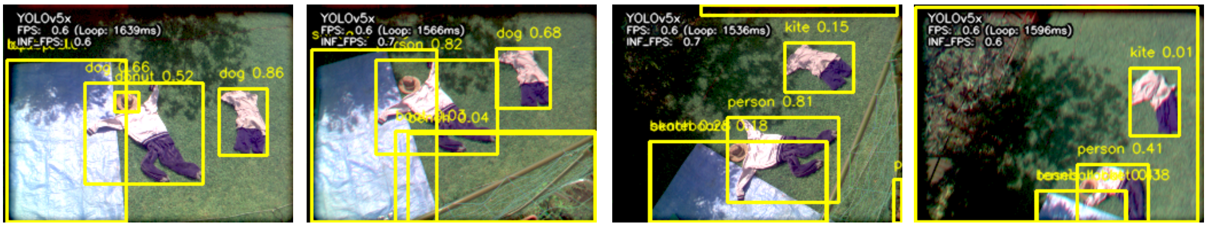

(a) (b) (c) (d)

Figure S8: Failure mode analysis of YOLOv5x on the Simulated SAR Scene. Detection results with all 80 MS COCO classes enabled, illustrating the root cause of low person-class AP reported in Section 4.5.2. The smaller target is predominantly misclassified as ”dog” with high confidence (a, b), while the larger simulated person is sporadically detected as ”person” with varying confidence (c, d). This behavior demonstrates the fundamental challenge for shape-based detectors when classifying atypical, non-human-like shapes as the person class—targets that represent significant deviations from typical postures in the MS COCO training data.

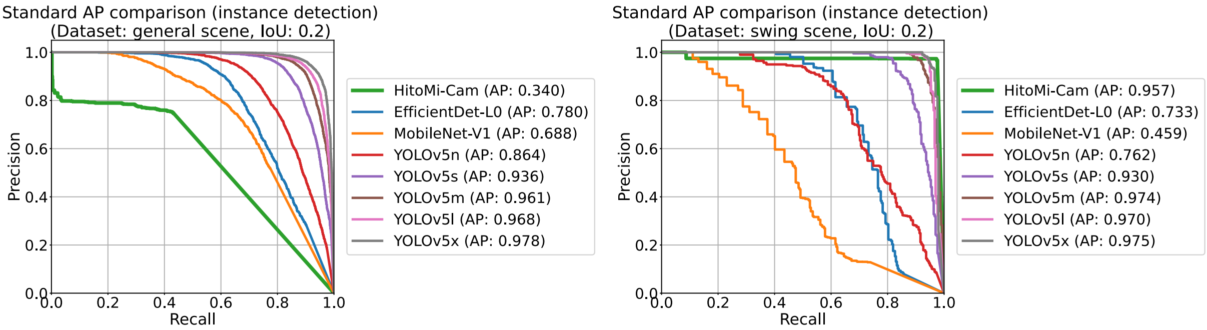

(a) General scene (b) Swing scene

Figure S9: PR curves of each model for General Scene and Swing Scene (IoU $\geq$ 0.2). This figure shows the PR curves of all compared models (*HitoMi-Cam*, YOLOv5 family, MobileNet-V1, and EfficientDet-L0). (a) For General Scene, the CNN-based models (especially the YOLOv5 family) significantly outperform *HitoMi-Cam*. (b) For Swing Scene, *HitoMi-Cam* achieves a high average precision (AP) and shows superiority over lightweight CNNs (such as YOLOv5n). The horizontal axis is recall, and the vertical axis is precision; the upper right quadrant shows higher performance.

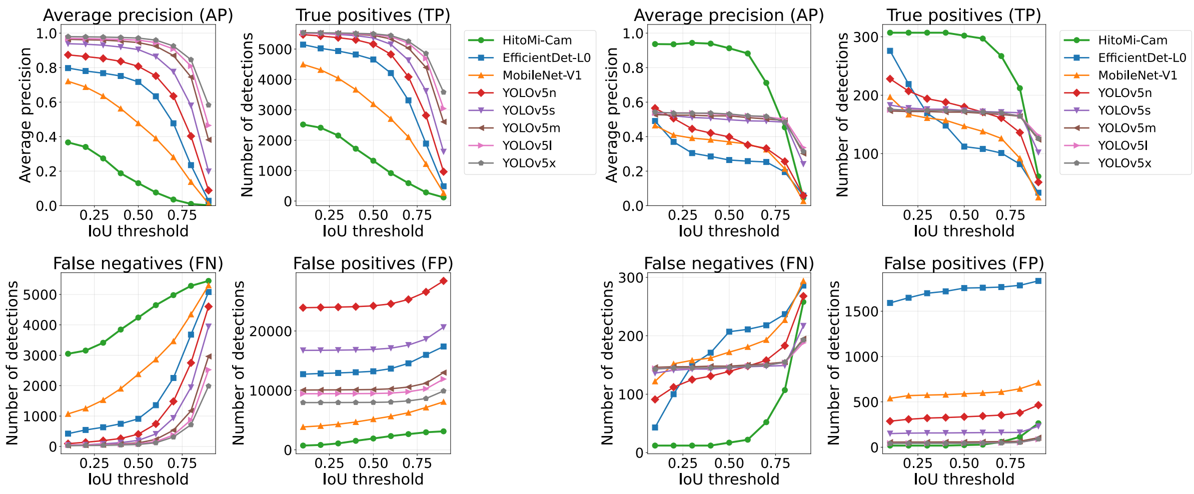

(a) General scene

(b) Simulated SAR scene

Figure S10: Transition of performance metrics with respect to IoU threshold changes in (a) General Scene and (b) Simulated SAR Scene. Average Precision (AP), True Positives (TP), False Negatives (FN), and False Positives (FP) are shown. The sharp drop in the TP rate for *HitoMi-Cam*, which was prominent for Swing Scene, is not clear in the other scenarios, and high performance is maintained even at IoU = 0.5, especially for Simulated SAR Scene.

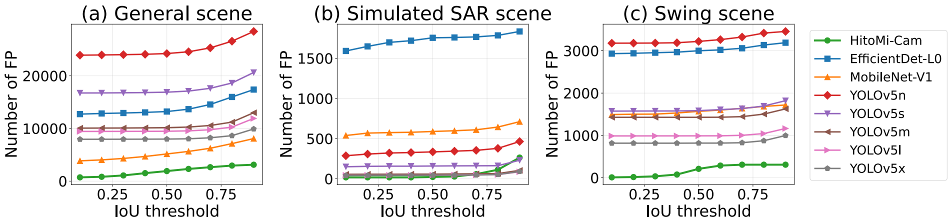

Figure S11: Number of False Positives (FP) versus the IoU threshold across all three scenarios. This figure plots the number of FPs for *HitoMi-Cam* and the compared CNN models (YOLOv5 family, MobileNet-V1, and EfficientDet-L0) as a function of the IoU threshold: (a) General Scene, (b) Simulated SAR Scene, and (c) Swing Scene. Because *HitoMi-Cam* is designed to output only candidates with a confidence of 1.0 based on physical detection, it does not generate numerous low-confidence candidates like the CNN models do. Consequently, it exhibits a consistently lower number of FPs in all scenarios.

Supplementary Videos

Video S1: Real-time operation demonstration on Raspberry Pi 5

This video demonstrates the real-time operation of four detection methods running on the Raspberry Pi 5 single-board computer: *HitoMi-Cam*, YOLOv5n, MobileNet-V1, and EfficientDet-L0. The video shows a parallel comparison in a challenging dynamic scene where the target undergoes extreme posture changes.

Key observations from the video:

- *HitoMi-Cam* operates at 23.2 fps and maintains stable detection throughout the sequence, continuously tracking the target despite rapid motion and atypical postures.
- YOLOv5n operates at 4.7 fps and shows intermittent detection instability for non-standard poses, despite its higher baseline accuracy in typical scenarios.
- MobileNet-V1 operates at 15.4 fps but exhibits occasional detection failures during extreme posture changes.

- EfficientDet-L0 operates at 7.6 fps and demonstrates moderate performance for this challenging scenario.

The video visually confirms that *HitoMi-Cam* achieves both high-speed processing and stable shape-agnostic detection on resource-constrained edge hardware, demonstrating its practical viability for real-time applications.
Video specifications:

- Format: MP4 (H.264)
- Resolution: 546 × 480 pixels
- Duration: 12 seconds
- File: Video_S1.mp4

Supplementary Tables

Table S1: Comparison of processing times across different sensor modes and resolutions. This table presents the detailed processing time data (mean ± standard deviation) from the preliminary experiments that informed the selection of the operational configuration used in this study. The results confirm that a resolution of 253 × 190 pixels captured in binning mode (corresponding to "2K_2" in the table) was the optimal configuration capable of achieving near-real-time performance (approx. 23.2 frames per second (fps); 43.1 ms total) on the Raspberry Pi 5 single-board computer (Raspberry Pi Ltd., Cambridge, UK). Consequently, this configuration was adopted for all subsequent evaluation experiments.

**Metric**	**4K_1**	**4K_2**	**4K_4**	**2K_1**	**2K_2**
Image resolution (pixels)	1014 × 760	507 × 380	253 × 190	507 × 380	253 × 190
Pre-processing (ms)					
Image acquisition	0.0 ± 0.0	0.0 ± 0.0	40.8 ± 11.3	0.0 ± 0.0	0.0 ± 0.0
RAW data conversion	10.9 ± 2.2	6.0 ± 0.4	6.1 ± 0.2	2.7 ± 0.7	2.3 ± 0.5
RAW reconstruction	23.0 ± 3.2	19.0 ± 2.9	16.8 ± 0.5	6.5 ± 1.4	6.0 ± 1.7
Bayer processing	0.0 ± 0.0	13.9 ± 0.6	3.7 ± 0.3	0.0 ± 0.0	3.8 ± 0.5
Luminance calculation	26.8 ± 3.7	5.9 ± 0.7	1.4 ± 0.1	11.4 ± 1.5	2.0 ± 0.7
Luminance reconstruction	29.9 ± 2.9	5.1 ± 0.9	1.5 ± 0.2	7.8 ± 1.2	2.5 ± 0.9
Subtotal: Pre-processing (ms)	115.8 ± 16.8	55.0 ± 6.0	30.1 ± 1.3	35.2 ± 5.7	17.8 ± 4.8
MLP Inference (ms)					
Data type conversion	6.9 ± 1.8	1.1 ± 0.2	0.3 ± 0.0	2.0 ± 0.4	0.5 ± 0.3
Data preparation	18.4 ± 3.0	3.9 ± 0.4	0.5 ± 0.0	4.9 ± 0.5	0.7 ± 0.3
Inference (Core)	173.6 ± 5.8	52.0 ± 5.1	8.6 ± 1.0	42.7 ± 1.3	9.8 ± 1.5
Subtotal: MLP Inference (ms)	228.0 ± 5.9	65.8 ± 5.2	11.9 ± 1.2	56.2 ± 1.3	13.4 ± 1.7
Post-processing (ms)					
Pseudo-color generation	73.6 ± 2.6	14.5 ± 1.1	3.0 ± 0.3	17.9 ± 1.7	4.4 ± 1.4
Result colorization	13.6 ± 1.5	4.4 ± 0.7	0.7 ± 0.1	3.1 ± 0.3	0.8 ± 0.1
Bounding box generation	86.7 ± 1.7	21.6 ± 0.7	5.3 ± 0.5	21.7 ± 0.7	5.5 ± 0.4
Subtotal: Post-processing (ms)	173.9 ± 5.9	40.5 ± 2.4	9.0 ± 0.8	42.8 ± 2.7	10.8 ± 1.9
Total Time per Frame (ms)	520.2 ± 9.5	182.1 ± 7.9	179.4 ± 26.4	135.2 ± 2.5	43.1 ± 3.1

Table S2: List of 41 labels used for MLP training. The table lists the 39 clothing categories and 2 background categories used to train the classifier. The clothing samples consist of common materials such as polyester, cotton, and wool and cover a wide range of colors. This table is adapted from the author's previous work.

Label Name [1]	Index	R-channel Intensity
Inorganic background	0	0
Plant background	34	255
P.Fluorescent-Yellow	1	53
P.Gray 2	2	245
P.White + C.White	3	67
P.Black + C.Black	4	204
P.Gray 1 + W.Gray 1 + C.Lt. Gray	5	145
P.Fluorescent-Orange	6	135
P.Blue + C.Blue	7	219
P.Yellow + C.Yellow	8	127
P.Navy 2	9	187
P.Navy 1 + C.Navy	10	72
P.Red + C.Red + W.Red	11	178
P.Green + C.Green	12	248
C.Gray 2	13	244
C.Gray 1	14	189
C.Purple + W.Navy	15	64
W.Black + W.Jet-Black	16	197
W.Yellow	17	128
W.Gray 2	18	65
W.Khaki + C.Lt. Olive	19	171
W.Blue	20	241
W.White	21	153
W.Lt. Blue + P.Lt. Blue + C.Lt. Blue	22	240
W.Green	23	188
P.Sax Blue	25	246
C.Lavender	26	236
P.Lt. Green + C.Bright Green	27	252
C.Light Beige + W.J. Sand Beige	28	233
W.G. 1 Beige + W.T. 2 Beige	29	227
W.T. 1 Ivory Beige	30	214
P.Lt. Pink + C.Baby Pink + C.Lt. Pink	35	251
W.G. 13 Cream Yellow	36	206
P.MC. Pink	38	253
P.Lt. Yellow + C.Lt. Yellow	39	243

Continued on next page

Table S2 – continued from previous page

Label Name [1]	Index	R-channel intensity
P.MC. Red	40	239
A. Orange + Glim. Orange	41	203
P.MC. Purple	43	247
W.J. 6 Magenta	45	221
C.Lt. Purple	46	232
P.Lt. Purple	47	249

[1] Key to abbreviations: P. = polyester, C. = cotton, W. = wool, T. = Toray (a Japanese synthetic fabric manufacturer), G. = gabardine, MC. = mixed color, J. = Josette (a linen-based fabric), A. = athletic, Glim. = glimmer, and Lt. = light.

Table S3: Detailed performance metrics of each model across all three scenarios with an IoU threshold of 0.2. This table shows all performance metrics (AP: Average Precision, Precision, Recall, F1 score, TP: True Positives, FP: False Positives, and FN: False Negatives) for all compared models in all three scenarios.

Scene	Model	AP	Precision	Recall	F1	TP	FP	FN
General Scene	HitoMi-Cam	0.3401	0.7458	0.4333	0.6394	2412	822	3154
	EfficientDet-L0	0.7803	0.2810	0.9024	0.5901	5023	12850	543
	MobileNet-V1	0.6876	0.5169	0.7752	0.7706	4315	4033	1251
	YOLOv5n	0.8641	0.1847	0.9754	0.4642	5429	23964	137
	YOLOv5s	0.9356	0.2476	0.9903	0.5520	5512	16748	54
	YOLOv5m	0.9612	0.3540	0.9923	0.6698	5523	10079	43
	YOLOv5l	0.9680	0.3686	0.9903	0.6545	5512	9442	54
	YOLOv5x	0.9784	0.4102	0.9941	0.6980	5533	7954	33
Simulated SAR Scene	HitoMi-Cam	0.9353	0.9446	0.9624	0.9710	307	18	12
	EfficientDet-L0	0.3697	0.1173	0.6865	0.2731	219	1648	100
	MobileNet-V1	0.4094	0.2269	0.5235	0.4338	167	569	152
	YOLOv5n	0.5056	0.4019	0.6489	0.5866	207	308	112
	YOLOv5s	0.5202	0.5345	0.5580	0.6244	178	155	141
	YOLOv5m	0.5243	0.7511	0.5392	0.6705	172	57	147
	YOLOv5l	0.5376	0.8028	0.5486	0.6807	175	43	144
	YOLOv5x	0.5363	0.8018	0.5455	0.6794	174	43	145
Swing Scene	HitoMi-Cam	0.9574	0.9450	0.9766	0.9605	292	17	7
	EfficientDet-L0	0.7330	0.0801	0.8562	0.1952	256	2940	43
	MobileNet-V1	0.4589	0.1283	0.7391	0.2594	221	1501	78
	YOLOv5n	0.7623	0.0838	0.9732	0.2061	291	3180	8
	YOLOv5s	0.9301	0.1568	0.9799	0.3476	293	1576	6
	YOLOv5m	0.9741	0.1722	0.9933	0.3620	297	1428	2
	YOLOv5l	0.9702	0.2299	0.9866	0.4361	295	988	4
	YOLOv5x	0.9755	0.2640	0.9799	0.4730	293	817	6

Table S4: Detailed performance metrics of each model in the Swing Scene. This table shows all the performance metrics (AP: Average Precision, TP: True Positives, FN: False Negatives, FP: False Positives) for all compared models in the Swing Scene, calculated by varying the IoU threshold in 0.1 increments.

Model	IoU Threshold	AP	TP	FN	FP
HitoMi-Cam	0.1	0.9991	299	0	10
	0.2	0.9574	292	7	17

Continued on next page

Table S4 – continued from previous page

Model	IoU Threshold	AP	TP	FN	FP
	0.3	0.8736	275	24	34
	0.4	0.6048	232	67	77
	0.5	0.1196	98	201	211
	0.6	0.0063	21	278	288
	0.7	0	0	299	309
	0.8	0	0	299	309
	0.9	0	0	299	309
EfficientDet-L0	0.1	0.7405	264	35	2932
	0.2	0.7330	256	43	2940
	0.3	0.6827	242	57	2954
	0.4	0.5176	229	70	2967
	0.5	0.4429	196	103	3000
	0.6	0.3835	175	124	3021
	0.7	0.2737	137	162	3059
	0.8	0.0640	61	238	3135
	0.9	0.0026	6	293	3190
MobileNet-V1	0.1	0.4767	229	70	1493
	0.2	0.4589	221	78	1501
	0.3	0.4429	218	81	1504
	0.4	0.3164	192	107	1530
	0.5	0.1759	153	146	1569
	0.6	0.1041	122	177	1600
	0.7	0.0494	82	217	1640
	0.8	0.0074	37	262	1685
	0.9	0.0001	3	296	1719
YOLOv5n	0.1	0.8124	292	7	3179
	0.2	0.7623	291	8	3180
	0.3	0.7057	289	10	3182
	0.4	0.4563	282	17	3189
	0.5	0.3351	250	49	3221
	0.6	0.2644	208	91	3263
	0.7	0.1816	147	152	3324
	0.8	0.0221	54	245	3417
	0.9	0.0015	15	284	3456
YOLOv5s	0.1	0.9346	295	4	1574
	0.2	0.9301	293	6	1576
	0.3	0.9212	292	7	1577
	0.4	0.9015	291	8	1578
	0.5	0.8730	284	15	1585
	0.6	0.7946	259	40	1610
	0.7	0.7158	235	64	1634
	0.8	0.4355	175	124	1694
	0.9	0.0387	48	251	1821
YOLOv5m	0.1	0.9745	297	2	1428
	0.2	0.9741	297	2	1428
	0.3	0.9740	297	2	1428
	0.4	0.9695	297	2	1428
	0.5	0.9694	297	2	1428
	0.6	0.9609	295	4	1430
	0.7	0.9127	281	18	1444
	0.8	0.6075	221	78	1504
	0.9	0.1728	98	201	1627

Continued on next page

Table S4 – continued from previous page

Model	IoU Threshold	AP	TP	FN	FP
YOLOv5l	0.1	0.9709	295	4	988
	0.2	0.9702	295	4	988
	0.3	0.9691	294	5	989
	0.4	0.9691	294	5	989
	0.5	0.9650	293	6	990
	0.6	0.9609	292	7	991
	0.7	0.9163	280	19	1003
	0.8	0.6931	239	60	1044
	0.9	0.2149	119	180	1164
YOLOv5x	0.1	0.9755	293	6	817
	0.2	0.9755	293	6	817
	0.3	0.9754	293	6	817
	0.4	0.9752	293	6	817
	0.5	0.9695	291	8	819
	0.6	0.9684	291	8	819
	0.7	0.9284	282	17	828
	0.8	0.6952	235	64	875
	0.9	0.1913	112	187	998
